# Protein-dependent CD4^+^ T cell specialization and CD8^+^ T cell-driven IL-2 production in HIV

**DOI:** 10.1016/j.isci.2026.115895

**Published:** 2026-04-27

**Authors:** Jernej Pušnik, Falko M. Heinemann, Andreas Heinold, Enrico Richter, Stefan Esser, Hendrik Streeck

**Affiliations:** 1 Institute of Virology, University Hospital Bonn, Bonn, Germany; 2 German Center for Infection Research (DZIF), partner site Bonn–Cologne, Braunschweig, Germany; 3 Institute for Transfusion Medicine, University Hospital Essen, Essen, Germany; 4 Institute for the Research on HIV and AIDS–associated Diseases Essen, University Duisburg–Essen, Essen, Germany; 5 HPSTD Clinic, University Hospital Essen, University Duisburg–Essen, Essen, Germany

**Keywords:** Molecular biology, Immunology

## Abstract

Chronic HIV infection alters antiviral T cell responses and shapes their functional profiles. We combined HLA peptide tetramers with functional stimulation assays to characterize HIV-specific CD4^+^ and CD8^+^ T cells targeting immunodominant epitopes in chronically infected individuals. CD4^+^ and CD8^+^ T cells were present at comparable frequencies but showed distinct functional programs. CD4^+^ T cells displayed helper-associated features and were often functionally unresponsive, whereas CD8^+^ T cells exhibited cytotoxic profiles and were a major source of IL-2. CD4^+^ T cell functions were protein dependent: p24-specific cells were more cytotoxic and responsive, whereas gp120-specific cells preferentially expressed CXCR5. CD8^+^ T cell functions, particularly IL-2 and IFN-γ production inversely associated with viral load and positively associated with CD4^+^ T cell counts and the CD4/CD8 ratio. Collectively, these findings show CD8^+^ T cells as major IL-2 producers and protein-specific CD4^+^ T cell specialization, informing HIV vaccine and cure strategies.

## Highlights

• HIV-specific CD4^+^ and CD8^+^ T cells show distinct functional profiles
• CD4^+^ T cell functions depend on HIV protein specificity
• CD8^+^ T cells are a major source of IL-2 production
• CD8^+^ T cell functions are inversely associated with disease progression

## Graphical abstract

**Figure:**
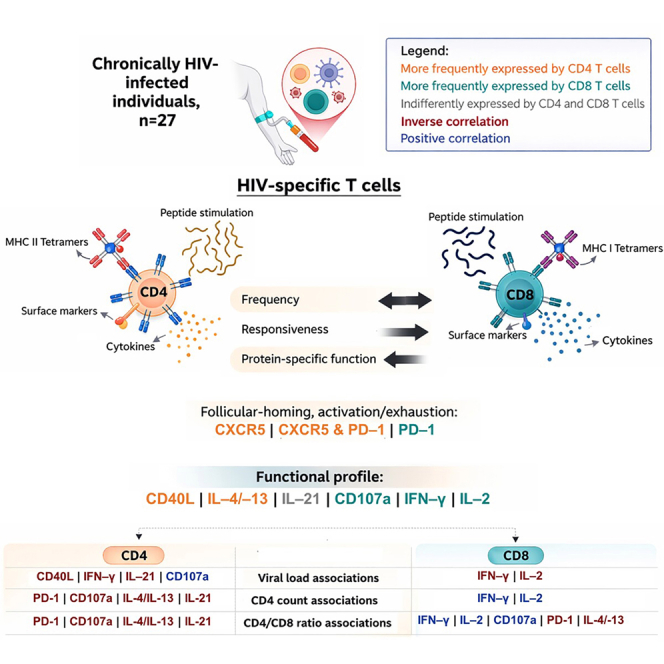

## Introduction

HIV infection profoundly disrupts T cell homeostasis, altering both the proportions and functions of CD4^+^ and CD8^+^ T cell subsets.^1^^,^^2^^,^^3^ During acute infection, up to 60% of memory CD4^+^ T cells are depleted, with preferential loss of HIV-specific cells continuing into the chronic phase of the disease.^1^^,^^2^^,^^3^^,^^4^ This depletion undermines immune control of viral replication, resulting in persistent antigenemia and chronic immune activation.^4^^,^^5^ Continuously activated CD4^+^ T cells become increasingly susceptible to HIV infection and activation-induced cell death, accelerating their depletion.^6^^,^^7^ Chronic activation also drives T cell exhaustion, a state marked by the expression of inhibitory receptors like PD-1 and a reduced ability to respond to HIV antigens.^7^^,^^8^

These and probably other, yet unknown mechanisms impair various functions of HIV-specific T cells. In the CD8^+^ T cell compartment, the predominant alterations include exhaustion-driven loss of cytotoxicity and polyfunctionality.^7^^,^^9^^,^^10^ Both CD4^+^ and CD8^+^ T cells show reduced production of IL-2, a cytokine crucial for the proliferation and survival of T cells. The same applies to the IFN-γ, a key molecule involved in the control of viral replication through activation of CD8^+^ T cells and macrophages.^1^^,^^11^^,^^12^^,^^13^^,^^14^ Also the frequency of IL-21-producing CD4^+^ T cells becomes decreased, compromising the activation and cytotoxic function of CD8^+^ T cells.^15^^,^^16^^,^^17^^,^^18^^,^^19^ Furthermore, HIV infection increases production of IL-4 in CD4^+^ T cells, shifting the balance between Th1 and Th2 responses in favor of the latter.^20^ This not only undermines the CD8^+^ T cell-mediated immune response but also contributes to the polyclonal B cell activation and hypergammaglobulinemia that are hallmarks of HIV-induced B cell dysregulation.^18^^,^^20^ Further compromising the B cell response, reduced expression of CD40L on CD4^+^ T cells and perturbations of the CXCR5^+^ T follicular helper (Tfh) cell compartment have been reported.^17^^,^^19^ Despite being particularly susceptible to HIV infection, Tfh cells expand due to the persistent antigenemia and show reduced levels of CD40L, IL–4, and IL-21 production.^21^^,^^22^ This hampers their ability to provide help to B cells during the germinal center reaction, causing anomalies in antibody production.^18^ CD8^+^ T cells can also express CXCR5 and access the germinal centers.^23^ In HIV infection, however, these follicular CD8^+^ T (Tfc) cells often express inhibitory receptors like PD-1 and show diminished cytotoxicity.^24^ Compromised cytotoxic function has also been observed for cytolytic CD4^+^ T cells that expand in the early phase of HIV infection and become exhausted due to persistent immune activation.^25^^,^^26^ Collectively, HIV causes multifaceted perturbations of T cell immunity, disrupting the balance between functional subsets and compromising their antiviral capacity.

Despite these impairments, HIV-specific T cells remain critical for viral control.^1^^,^^10^ Certain HLA class I alleles have been linked to superior CD8^+^ T cell responses, resulting in lower viral loads and slower disease progression.^27^^,^^28^ Also, subsets of HIV-specific CD4^+^ T cells, particularly those producing IL-21, have been associated with improved CD8^+^ T cell response and viral control.^15^^,^^16^ While these findings underscore the critical role of T cell responses in HIV infection, it remains unclear which factors shape the functions of HIV-specific CD4^+^ and CD8^+^ T cells and how their altered functional profiles differ. Previous studies suggested that the functions of T cells are dictated by antigen availability in HIV infection.^29^ Moreover, different HIV proteins seem to have different capacities to induce T-cell responses. For example, the p24 protein was found to be superior in inducing HIV-specific T cell responses^30^^,^^31^^,^^32^^,^^33^ due to its high abundance,^34^ stability,^35^ and conservation.^36^ Another factor possibly driving the function of T cells is the crosstalk between CD4^+^ and CD8^+^ T cells, since CD4^+^ T cells play a crucial role in the generation of long-lasting memory CD8^+^ T cells and in sustaining their function.^7^^,^^37^^,^^38^^,^^39^^,^^40^^,^^41^^,^^42^ Whether effective CD4^+^ help requires recognition of the same protein as CD8^+^ T cells remains debated. While overlapping CD4^+^ and CD8^+^ targets have been described for HIV, influenza, and SARS-CoV-2,^28^^,^^31^^,^^32^^,^^43^^,^^44^^,^^45^ other viruses such as vaccinia show discordant targeting patterns.^46^^,^^47^

In summary, HIV infection profoundly perturbs T cell function, yet HIV-specific T cell responses remain linked to viral control. However, how these altered functional profiles of HIV-specific CD4^+^ and CD8^+^ T cells compare, and which factors shape these profiles, has not been systematically investigated. Here, we compared the frequencies, follicular homing potential, and functional profiles of CD4^+^ and CD8^+^ T cells specific for immunodominant HIV epitopes in 27 chronically infected, treatment-na-ve individuals. We further examined the relationships between functional subsets of HIV-specific CD4^+^ and CD8^+^ T cells, their dependence on protein specificity, and their associations with key markers of disease progression, including plasma viral load, CD4^+^ T cell count, and the CD4/CD8 ratio.

## Results

### Frequencies of CD4^+^ and CD8^+^ T cells targeting immunodominant epitopes of the HIV proteome

**Figure 1:**
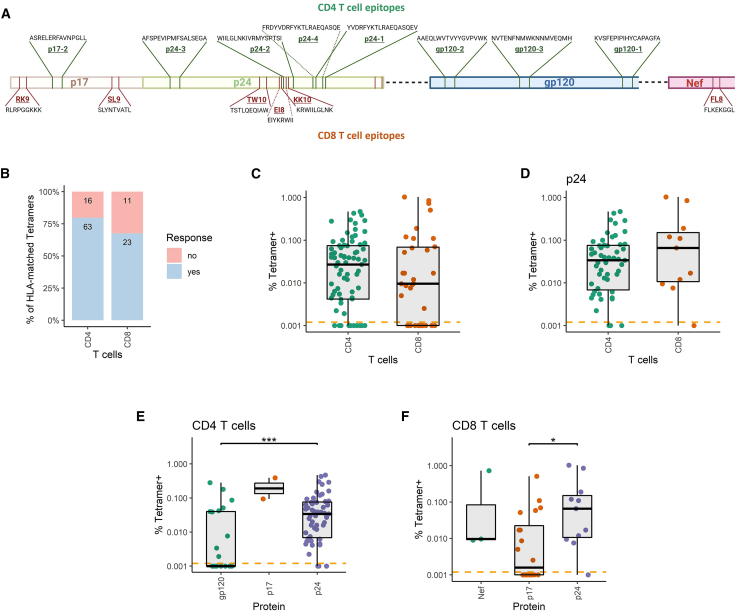
Frequency of CD4^+^ and CD8^+^ T cells specific for immunodominant HIV epitopes in peripheral blood of treatment-naive individuals in the chronic phase of HIV infection (A) HIV proteome mapping of the CD4^+^ and CD8^+^ T cell epitopes present on the synthesized HLA tetramers. Different proteins appear as bands of distinct colors. CD4^+^ T cell epitopes are presented above the protein bands, and CD8^+^ T cell epitopes are below the bands. For each epitope, the approximate location within the protein sequence is shown along with its name and full sequence. (B) Proportions of detectable responses against the synthesized tetramers in HLA-matched individuals for CD4^+^ and CD8^+^ T cells. The data are presented as stacked bar plots with a noted number of responsive and unresponsive matched tetramer-individual combinations. (C) Frequency of tetramer-binding cells as a percentage of CD4^+^ or CD8^+^ T cell populations. The dashed line represents the positivity cutoff. The following numbers of matched tetramer-individual combinations were included in each group: *n*(CD4) = 79, *n*(CD8) = 34. (D) Frequency of cells specific for tetramers presenting p24 epitopes as a percentage of CD4^+^ or CD8^+^ T cell populations. The dashed line represents the positivity cutoff. The following numbers of matched tetramer-individual combinations were included in each group: *n*(CD4) = 55, *n*(CD8) = 11. (E) Frequency of CD4^+^ T cells specific for tetramers presenting epitopes of different proteins (gp120, p17, and p24) as a percentage of the parent population. The dashed line represents the positivity cutoff. (F) Frequency of CD8^+^ T cells specific for tetramers presenting epitopes of different proteins (Nef, p17, and p24) as a percentage of the parent population. The dashed line represents the positivity cutoff. The following numbers of matched tetramer-individual combinations were included in each group: *n*(gp120) = 23, *n*(p17) = 2, *n*(p24) = 55 for (E), and *n*(Nef) = 3, *n*(p17) = 20, *n*(p24) = 11 for (F). In (C), (D), (E), and (F), the data are displayed as boxplots, indicating the range, first quartile, third quartile, median, and individual data points. For comparisons between two groups, the Mann-Whitney test was used. For comparisons among three groups, the Kruskal-Wallis test was performed, followed by pairwise Mann-Whitney tests with Holm’s correction for multiple comparisons. All tests were two sided. Statistical significance is indicated by the following annotations: ∗*p* < 0.05, ∗∗*p* < 0.01, ∗∗∗*p* < 0.001, ∗∗∗∗*p* < 0.0001.

To compare the properties of HIV-specific CD4^+^ and CD8^+^ T cells targeting different protein regions, we collected peripheral blood samples of 27 treatment-naive chronically HIV-infected individuals. Based on our previous findings that highlight the prevalence of specific HLA alleles among HIV-infected individuals in Europe and North America, as well as the frequency of T cell responses to particular epitopes,^28^^,^^31^^,^^32^^,^^45^ we synthesized 15 HLA class II and 7 HLA class I tetramers covering compatible HLA-peptide combinations most frequently recognized by T cells of HIV-infected individuals (Table 1; Figure 1A). Isolated peripheral blood mononuclear cells (PBMCs) were then labeled with fluorescent HLA-peptide tetramers and stimulated with respective peptides (full gating strategy in Figure S1). The expression of surface proteins and cytokines in response to stimulation was monitored by flow cytometry.

**Table 1:** List of HLA-peptide combinations synthesized for this study

HLA class I/II	Peptide	Sequence
A∗03:01	RK9	RLRPGGKKK
A∗02:01	SL9	SLYNTVATL
B∗07:02	GL9	GPGHKARVL
B∗08:01	FL8	FLKEKGGL
B∗08:01	EI8	EIYKRWII
B∗27:05	KK10	KRWIILGLNK
B∗57:01	TW10	TSTLQEQIAW
DRB1∗07:01	p24-1	YVDRFYKTLRAEQASQEV
DRB1∗07:01	p24-4	FRDYVDRFYKTLRAEQASQE
DRB1∗15:01	p24-2	WIILGLNKIVRMYSPTSI
DRB1∗15:01	p24-3	AFSPEVIPMFSALSEGA
DRB1∗15:01	gp120-2	AAEQLWVTVYYGVPVWK
DRB1∗15:01	gp120-3	NVTENFNMWKNNMVEQMH
DRB1∗01:01	p24-1	YVDRFYKTLRAEQASQEV
DRB1∗01:01	p24-4	FRDYVDRFYKTLRAEQASQE
DRB1∗01:01	gp120-1	KVSFEPIPIHYCAPAGFA
DRB1∗13:01	p17-2	ASRELERFAVNPGLL
DRB1∗13:01	p24-1	YVDRFYKTLRAEQASQEV
DRB1∗13:01	p24-2	WIILGLNKIVRMYSPTSI
DRB1∗11:01	p24-1	YVDRFYKTLRAEQASQEV
DRB1∗11:01	p24-4	FRDYVDRFYKTLRAEQASQE
DRB1∗03:01	p24-2	WIILGLNKIVRMYSPTSI

The names of the HLA allele and fused peptides with their full amino acid sequence are given within the same row.

Our findings demonstrate that 80% of the HLA-II-matched tetramer-individual combinations exhibited detectable tetramer-specific CD4^+^ T cell responses, while 68% of HLA-I-matched combinations had detectable tetramer-specific CD8^+^ T cell responses (Figure 1B). The frequency of CD4^+^ and CD8^+^ T cells specific for the tested tetramers was not significantly different when comparing both subsets (*p* = 0.49) (Figure 1C). The same was true when comparing only CD4^+^ and CD8^+^ T cell responses targeting the same region, in this case, p24 protein (*p* = 0.32) (Figure 1D). To address the immunogenicity of different proteins, we next compared the frequencies of tetramer-specific T cells recognizing epitopes present in distinct HIV proteins (gp120, p17, and p24 for CD4^+^ T cells and Nef, p17, and p24 for CD8^+^ T cells). The frequencies of p24-specific CD4^+^ T cells were significantly higher than those of gp120-specific but not p17-specific CD4^+^ T cells (*p* < 0.001) (Figure 1E). For CD8^+^ T cells, more tetramer-specific cells were observed for p24 epitopes compared to p17 epitopes (*p* < 0.05) but not Nef epitopes (Figure 1F). Observations for p17-specific CD4^+^ T cells and Nef-specific CD8^+^ T cells should be interpreted with caution, as the analysis is limited by the small number of data points.

In summary, our data demonstrate comparable frequencies of cells specific for the tested HIV epitopes among the CD4^+^ and CD8^+^ subsets. The p24 epitopes were particularly immunogenic for both T cell subsets, distinguishing them in terms of their specificity for particular HIV proteins.

### Accessibility of B cell follicles by CD4^+^ and CD8^+^ T cells targeting immunodominant epitopes of the HIV proteome

**Figure 2:**
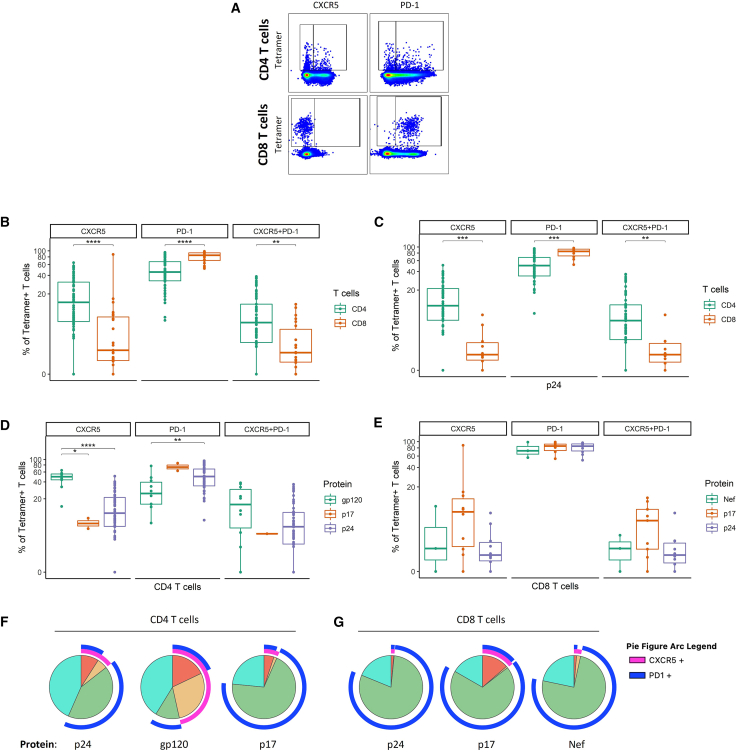
Expression of CXCR5 and PD-1 on CD4^+^ and CD8^+^ T cells specific for immunodominant HIV epitopes (A) Representative flow cytometry pseudocolor plots with gating for the assessment of CXCR5 and PD-1 expression on tetramer-specific T cells. For the detailed gating strategy, see Figure S1. (B) Frequency of cells expressing CXCR5, PD-1, or both as a percentage of tetramer-binding CD4^+^ or CD8^+^ T cell populations. The following numbers of matched tetramer–individual combinations were included in each group: *n*(CD4) = 63, *n*(CD8) = 23. (C) Proportion of cells expressing CXCR5, PD-1, or both as a percentage of tetramer-binding p24-specific CD4^+^ or CD8^+^ T cell populations. The following numbers of matched tetramer–individual combinations were included in each group: *n*(CD4) = 51, *n*(CD8) = 10. (D) Proportion of cells expressing CXCR5, PD-1 or both as a percentage of tetramer-binding CD4^+^ T cells of different protein specificities (gp120, p17, and p24). (E) Proportion of cells expressing CXCR5, PD-1 or both as a percentage of tetramer-binding CD8^+^ T cells of different protein specificities (Nef, p17, and p24). The following numbers of matched tetramer-individual combinations were included in each group: *n*(gp120) = 10, *n*(p17) = 2, *n*(p24) = 51 for (D) and *n*(Nef) = 3, *n*(p17) = 10, *n*(p24) = 10 for (E). (F) Pie charts demonstrating the co–expression of CXCR5 and PD-1 on tetramer-binding CD4^+^ T cells of different protein specificities (gp120, p17, and p24). (G) Pie charts demonstrating the co–expression of CXCR5 and PD-1 on tetramer-binding CD8^+^ T cells of different protein specificities (Nef, p17, and p24). Each slice represents a distinct subset regarding the co-expression of CXCR5 and PD-1. Arcs surrounding the pie chart indicate which proteins are expressed by a certain subset. Differences between the pie charts were assessed by the permutation test. The following numbers of matched tetramer-individual combinations were included in each group: *n*(gp120) = 10, *n*(p17) = 2, *n*(p24) = 51 for (F), and *n*(Nef) = 3, *n*(p17) = 10, *n*(p24) = 10 for (G). In (B)–(E), the data are displayed as boxplots, indicating the range, first quartile, third quartile, median, and individual data points. For comparisons between two groups, the Mann-Whitney test was used. For comparisons among three groups, the Kruskal-Wallis test was performed, followed by pairwise Mann-Whitney tests with Holm’s correction for multiple comparisons. All tests were two sided. Statistical significance is indicated by the following annotations: ∗*p* < 0.05, ∗∗*p* < 0.01, ∗∗∗*p* < 0.001, ∗∗∗∗*p* < 0.0001.

CD4^+^ T cell participation in germinal center reaction is indispensable for the production of antibodies against HIV, whereas CD8^+^ T cells that access B cell follicles contribute to reducing the viral reservoir.^48^^,^^49^^,^^50^ We next compared the expression of follicular-homing markers, CXCR5 and PD-1, on HIV-specific CD4^+^ and CD8^+^ T cells and assessed whether protein specificity influenced their expression (Figure 2A) (full gating strategy in Figure S1).

Our findings showed significantly higher frequencies of CXCR5^+^ expression on tetramer-specific CD4^+^ T cells compared to tetramer-specific CD8^+^ T cells (*p* < 0.0001), while PD-1 expression was more frequent on tetramer-specific CD8^+^ T cells (*p* < 0.0001). Moreover, CD4^+^ T cells had a higher proportion of CXCR5^+^PD-1^+^ cells compared to CD8^+^ T cells (*p* < 0.01) (Figure 2B). Similar was observed for CD4^+^ and CD8^+^ T cells targeting the same region (p24), where tetramer-specific CD4^+^ T cells showed increased frequencies of CXCR5^+^ and CXCR5^+^PD-1^+^ cells (*p* < 0.001 and *p* < 0.01), whereas PD-1^+^ cells were more frequent among tetramer-specific CD8^+^ T cells (*p* < 0.001) (Figure 2C). When analyzing the impact of HIV protein specificity, a higher proportion of CXCR5^+^ cells was observed among tetramer-specific CD4^+^ T cells recognizing gp120 compared to p17 (*p* < 0.05) and p24 (*p* < 0.0001) epitopes. PD-1^+^ cells were more frequent among p24-specific CD4^+^ T cells than among gp120-specific cells (*p* < 0.01). No significant differences were found for CXCR5^+^PD-1^+^ cells (Figure 2D). For tetramer-specific CD8^+^ T cells, there were no significant differences in the frequency of CXCR5 and PD-1-expressing cells comparing different protein specificities (Figure 2E). Observations for p17-specific CD4^+^ T cells and Nef-specific CD8^+^ T cells should be interpreted with caution, as the analysis is limited by the small number of data points. Next, we assessed polyfunctionality using SPICE software,^51^ with permutation testing, which evaluates differences across the entire distribution of marker expression patterns rather than comparing individual markers or specific marker combinations. We observed significantly different expression profiles of CXCR5 and PD-1 among tetramer-specific CD4^+^ T cells specific for gp120, p17, and p24 proteins (*p* < 0.0001 for all comparisons) (Figure 2F) but not in the case of tetramer-specific CD8^+^ T cells (Figure 2G).

Taken together, HIV-specific CD4^+^ T cells more frequently express CXCR5 alone or in combination with PD-1 and less frequently express only PD-1 compared to HIV-specific CD8^+^ T cells, even when targeting the same protein. In the case of CD4^+^ T cells, these expressions vary by HIV protein specificity.

### Functional profiles of CD4^+^ and CD8^+^ T cells targeting immunodominant epitopes of the HIV proteome

**Figure 3:**
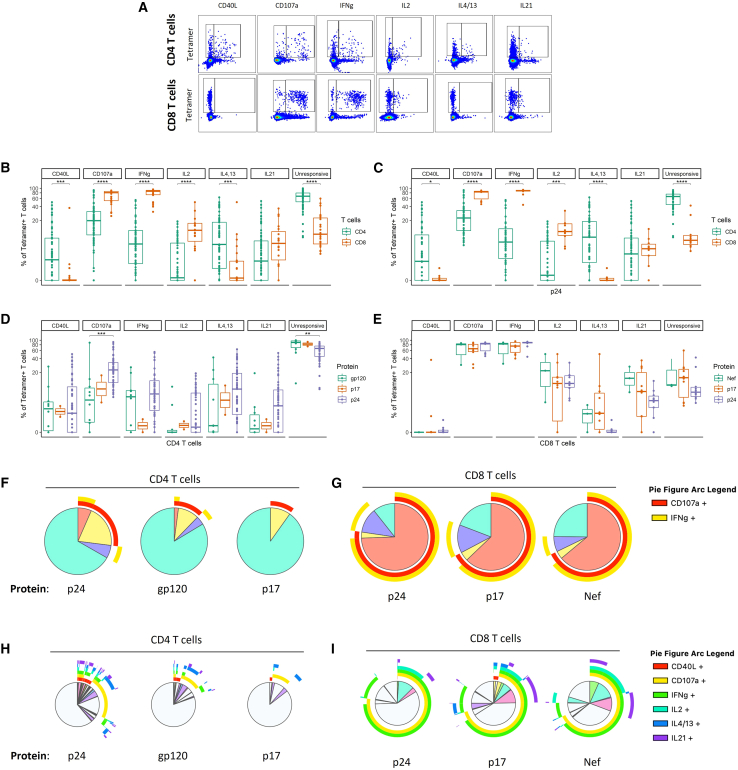
Functional profile of CD4^+^ and CD8^+^ T cells specific for immunodominant HIV epitopes (A) Representative flow cytometry pseudocolor plots with gating for the assessment of CD40L, CD107a, IFN-γ, IL-2, IL-4/-13, and IL-21 expression on tetramer-specific T cells. For the detailed gating strategy, see Figure S1. (B) Proportion of tetramer–binding cells expressing CD40L, CD107a, IFN-γ, IL-2, IL-4/-13, IL-21, or none of these proteins as a percentage of tetramer-binding CD4^+^ or CD8^+^ T cell populations. The following numbers of matched tetramer-individual combinations were included in each group: *n*(CD4) = 63, *n*(CD8) = 23. (C) Proportion of cells expressing CD40L, CD107a, IFN-γ, IL-2, IL-4/-13, IL-21, or none of these proteins as a percentage of tetramer-binding p24-specific CD4^+^ or CD8^+^ T cell populations. The following numbers of matched tetramer-individual combinations were included in each group: *n*(CD4) = 51, *n*(CD8) = 10. (D) Proportion of cells expressing CD40L, CD107a, IFN-γ, IL-2, IL-4/-13, IL-21, or none of these proteins as a percentage of tetramer-binding CD4^+^ T cells of different protein specificities (gp120, p17, and p24). (E) Proportion of cells expressing CD40L, CD107a, IFN-γ, IL-2, IL-4/-13, IL-21, or none of these proteins as a percentage of tetramer-binding CD8^+^ T cells of different protein specificities (Nef, p17, and p24). The following numbers of matched tetramer-individual combinations were included in each group: *n*(gp120) = 10, *n*(p17) = 2, *n*(p24) = 51 for (D) and *n*(Nef) = 3, *n*(p17) = 10, *n*(p24) = 10 for (E). (F) Pie charts demonstrating the co–expression of CD107a and IFN-γ on tetramer–binding CD4^+^ T cells of different protein specificities (gp120, p17, and p24). (G) Pie charts demonstrating the co–expression of CD107a and IFN-γ on tetramer–binding CD8^+^ T cells of different protein specificities (Nef, p17, and p24). Each slice represents a distinct subset regarding the co–expression of CD107a and IFN-γ. Arcs surrounding the pie chart indicate which proteins are expressed by a certain subset. The following numbers of matched tetramer-individual combinations were included in each group: *n*(gp120) = 10, *n*(p17) = 2, *n*(p24) = 51 for (F), and *n*(Nef) = 3, *n*(p17) = 10, *n*(p24) = 10 for (G). (H) Pie charts demonstrating the co–expression of CD40L, CD107a, IFN-γ, IL-2, IL-4/-13, and IL-21 on tetramer-binding CD4^+^ T cells of different protein specificities (gp120, p17, and p24). (I) Pie charts demonstrating the co–expression of CD40L, CD107a, IFN-γ, IL-2, IL-4/-13, and IL-21 on tetramer-binding CD8^+^ T cells of different protein specificities (Nef, p17, and p24). Each slice represents a distinct subset regarding the co-expression of CD40L, CD107a, IFN-γ, IL-2, IL-4/-13, and IL-21. Arcs surrounding the pie chart indicate which proteins are expressed by a certain subset. The following numbers of matched tetramer-individual combinations were included in each group: *n*(gp120) = 10, *n*(p17) = 2, *n*(p24) = 51 for (H) and *n*(Nef) = 3, *n*(p17) = 10, *n*(p24) = 10 for (I). Differences between the pie charts were assessed by the permutation test. In (B)–(E), the data are displayed as boxplots, indicating the range, first quartile, third quartile, median, and individual data points. For comparisons between two groups, the Mann-Whitney test was used. For comparisons among three groups, the Kruskal-Wallis test was performed, followed by pairwise Mann-Whitney tests with Holm’s correction for multiple comparisons. All tests were two-sided. Statistical significance is indicated by the following annotations: ∗*p* < 0.05, ∗∗*p* < 0.01, ∗∗∗*p* < 0.001, ∗∗∗∗*p* < 0.0001.

HIV infection profoundly disrupts T cell functions critical for antiviral immunity.^1^^,^^11^^,^^12^^,^^13^^,^^14^^,^^15^^,^^16^^,^^17^^,^^18^^,^^19^^,^^20^^,^^21^^,^^22^ To determine how these perturbations affect the relative contributions of CD4^+^ and CD8^+^ T cells to key antiviral functions, we compared the functional profiles of HIV-specific CD4^+^ and CD8^+^ T cells, assessing the expression of CD40L, CD107a, IFN-γ, IL-2, IL-4/-13, and IL-21 (Figure 3A). Using HLA-peptide tetramers in conjunction with peptide stimulation enabled us to distinguish between responsive and unresponsive HIV-specific cells.

Our data demonstrate an increased frequency of CD40L^+^ (*p* < 0.001) and IL-4/-13^+^ (*p* < 0.001) cells among tetramer-specific CD4^+^ T cells compared to CD8^+^ T cells. Conversely, CD107a (*p* < 0.0001), IFN-γ (*p* < 0.0001), and IL-2 (*p* < 0.0001) were more frequently induced in tetramer-specific CD8^+^ T cells. Of note, a higher percentage of cells that did not respond to stimulation with any of the analyzed functions was observed in the tetramer-specific CD4^+^ T cell population (*p* < 0.0001) (Figure 3B). Focusing on p24-specific T cells, CD4^+^ T cells more frequently expressed CD40L (*p* < 0.05) and IL-4/-13 (*p* < 0.0001), while CD8^+^ T cells more frequently expressed CD107a (*p* < 0.0001), IFN-γ (*p* < 0.0001), and IL-2 (*p* < 0.001). Again, there was a higher proportion of cells not responding to stimulation in the CD4^+^ T cell subset (*p* < 0.0001) (Figure 3C). Further examining the role of protein specificity in the functional profile of tetramer–specific T cells, we observed a higher frequency of CD107a^+^ (*p* < 0.001) and stimulation-responsive cells (*p* < 0.01) among p24-specific CD4^+^ T cells compared to gp120-specific CD4^+^ T cells (Figure 3D). No significant differences in frequencies of tetramer-specific cells exerting the assessed functions were observed among CD8^+^ T cells comparing different protein specificities (Figure 3E). Observations for p17-specific CD4^+^ T cells and Nef-specific CD8^+^ T cells should be interpreted with caution, as the analysis is limited by the small number of data points. To evaluate the impact of protein specificity on the cytotoxic potential of T cells, we assessed the combinatorial expression of CD107a and IFN-γ on tetramer-specific T cells targeting different proteins. This was done using SPICE software^51^ with permutation testing. CD4^+^ T cells targeting p24-derived epitopes had significantly different expression profiles compared to those targeting gp120 or p17-derived peptides (*p* < 0.01) (Figure 3F), while no significant differences were observed for CD8^+^ T cells (Figure 3G). Similarly, we investigated the polyfunctionality, in terms of CD40L, CD107a, IFN-γ, IL-2, IL-4/-13, and IL-21 expression, of T cells with different protein specificities and found a significantly different profile for p24-specific CD4^+^ T cells compared to gp120- or p17-specific CD4^+^ T cells (*p* < 0.01) (Figure 3H). Polyfunctional profiles of tetramer-specific CD8^+^ T cells did not depend on the protein targeting (Figure 3I).

In summary, our data demonstrate distinct functional profiles of HIV-specific CD4^+^ and CD8^+^ T cells regardless of protein targeting. CD4^+^ T cells more frequently expressed CD40L and IL-4/-13, while CD8^+^ T cells more frequently expressed CD107a, IFN-γ, and IL-2 and responded more consistently to stimulation. Remarkably, HIV-specific CD8^+^ T cells showed a higher proportion of cells that responded to stimulation with any of the analyzed functions. Notably, protein specificity shapes CD4^+^ T cell function, with p24-specific cells showing enhanced responsiveness and cytotoxic potential.

### Correlations between the functional traits of CD4^+^ and CD8^+^ T cells targeting immunodominant epitopes of the HIV proteome and associations with the HIV viral load

Since T cell function during HIV infection can be influenced by antigenemia,^29^^,^^52^^,^^53^ we analyzed correlations between the frequencies of tetramer-bindng HIV-specific T cells expressing functional markers and viral load.

**Figure 4:**
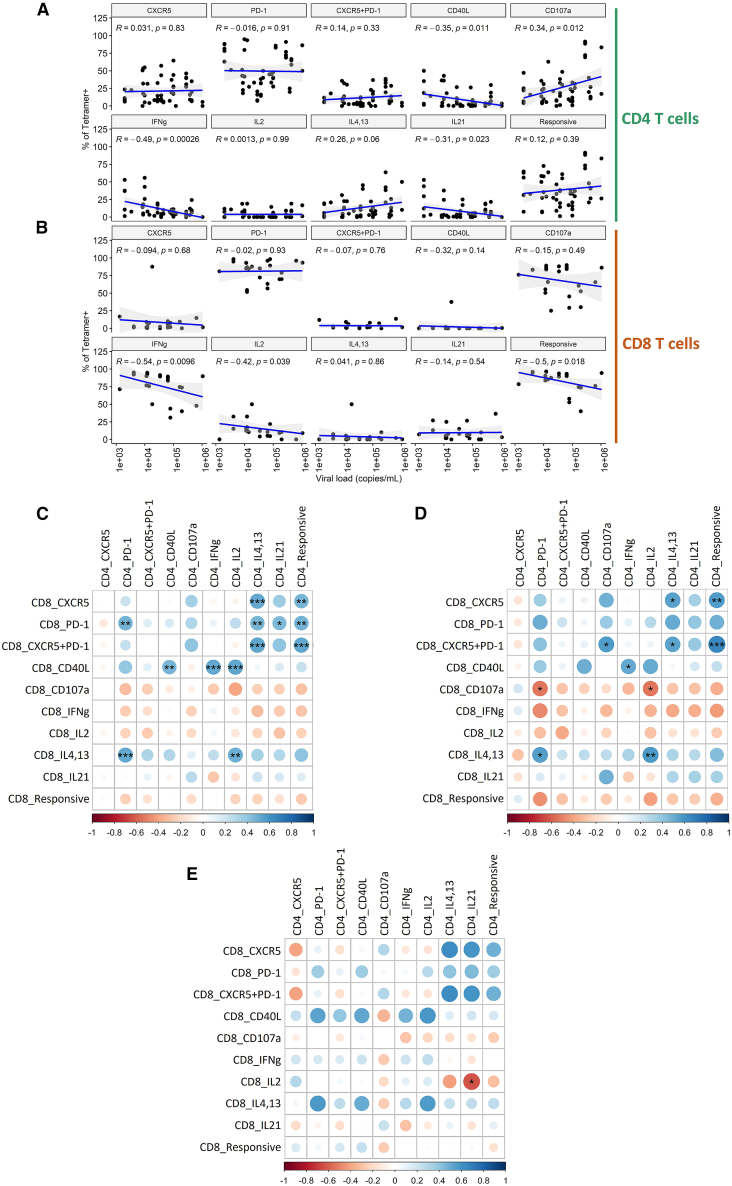
Correlations between the HIV viral load, phenotype, and functions of CD4^+^ and CD8^+^ T cells specific for immunodominant HIV epitopes (A) Scatterplots demonstrating correlations between the viral load and proportions of tetramer-specific CD4^+^ T cells expressing CXCR5, PD-1, CD40L, CD107a, IFN-γ, IL-2, IL-4/-13, IL-21, or any of these proteins. (B) Scatterplots demonstrating correlations between the viral load and proportions of tetramer-specific CD8^+^ T cells expressing CXCR5, PD-1, CD40L, CD107a, IFN-γ, IL-2, IL-4/-13, IL-21, or any of these proteins. The 95% confidence intervals around the line of best fit are displayed as shading. The r and *p* values are given for each line. The strength of correlations was assessed by a two–sided Spearman’s correlation test with Bonferroni’s correction for multiple testing. The following numbers of matched tetramer-individual combinations were included in each group: *n* = 63 for (A) and *n* = 23 for (B**)**. (C) Correlations between the frequencies of tetramer-specific CD4^+^ and CD8^+^ T cells exerting different phenotypes and functions are presented as a correlation matrix. The strength of a correlation (Spearman’s correlation coefficient) is depicted by the size and color of the circle; significance is indicated by asterisks. (D) Correlation matrix equivalent to that in (C), but only with pairs of CD4-CD8^+^ T cell responses targeting different proteins observed within the same individuals. (E) Correlation matrix equivalent to that in (C), but only with pairs of CD4-CD8^+^ T cell responses targeting the same (p24) protein. The strength of correlations was assessed by a two-sided Spearman’s correlation test with Bonferroni’s correction for multiple testing. The following numbers of CD4-CD8^+^ response pairs found within the same individual were included in each group: *n* = 68 for (C), *n* = 42 for (D), and *n* = 24 for (E). Statistical significance is indicated by the following annotations: ∗*p* < 0.05, ∗∗*p* < 0.01, ∗∗∗*p* < 0.001, ∗∗∗∗*p* < 0.0001.

Our data demonstrate that tetramer-specific CD4^+^ T cells expressing CD40L (r = −0.4, *p* < 0.05), IFN-γ (r = −0.5, *p* < 0.001), and IL-21 (r = −0.3, *p* < 0.05) inversely correlate with viral load in chronically infected individuals. On the contrary, the expression of CD107a (r = 0.3, *p* < 0.05) was positively correlated with the viral load (Figure 4A). In the case of the tetramer-specific CD8^+^ T cells, the expression of IFN-γ (r = −0.5, *p* < 0.01) and IL-2 (r = −0.4, *p* < 0.05) was inversely correlated with the viral load. The same was also observed for all stimulation-responsive tetramer-specific CD8^+^ T cells (r = −0.5, *p* < 0.05) (Figure 4B).

Given the dependence of CD8^+^ T cells on CD4^+^ T cell help in chronic viral infections,^7^^,^^41^^,^^42^ we next correlated the frequencies of functionally distinct tetramer-specific CD4^+^ and CD8^+^ T cells found within the same individuals. Our findings revealed positive correlations between the frequencies of tetramer-specific CD4^+^ T cells expressing IL-4/-13 and CD8^+^ T cells expressing PD-1 or/and CXCR5 (0.4 < r < 0.6, *p* < 0.01). Equivalent associations were observed for all stimulation-responsive tetramer-specific CD4^+^ T cells (0.4 < r < 0.6, *p* < 0.01). The expression of CD40L on tetramer-specific CD8^+^ T cells was correlated with tetramer-specific CD4^+^ T cells expressing CD40L, IFN-γ, or IL-2 (0.4 < r < 0.6, *p* < 0.01), and the expression of IL-4/-13 by CD8^+^ T cells was correlated with PD-1 or IL-2 expressing CD4^+^ T cells (0.4 < r < 0.6, *p* < 0.01) (Figure 4C). To investigate whether protein co-targeting might play a role in the delivery of CD4^+^ T cell help to CD8^+^ T cells, we performed the same correlation analysis but focused either on responses targeting the same or distinct proteins. We observed a high degree of similarity between all three correlation matrices. There was no indication that tetramer-specific CD4^+^ and CD8^+^ T-cell responses correlate better when targeting epitopes within the same protein (p24) compared to different proteins. On the contrary, nine significant correlations were observed for different proteins (Figure 4D) and only one for targeting the same protein (Figure 4E).

Overall, while HIV-specific T cells exhibiting distinct effector functions correlate differently with viral replication, IFN-γ expression by both CD4^+^ and CD8^+^ T cells consistently inversely correlates with viral load. Functional associations between HIV-specific CD4^+^ and CD8^+^ T cells are evident, but protein co-targeting does not appear to be a major determinant.

### Correlations between the functional traits of CD4^+^ and CD8^+^ T cells targeting immunodominant epitopes of the HIV proteome, CD4^+^ T cell count, and the CD4^+^/CD8^+^ T cell ratio

Given the associations observed between plasma viral load and functional traits of tetramer-defined HIV-specific T cell subsets, we next investigated how these subsets relate to further clinical immunological parameters commonly used to monitor HIV disease progression, including absolute CD4^+^ T cell counts and the CD4^+^/CD8^+^ T cell ratio.

**Figure 5:**
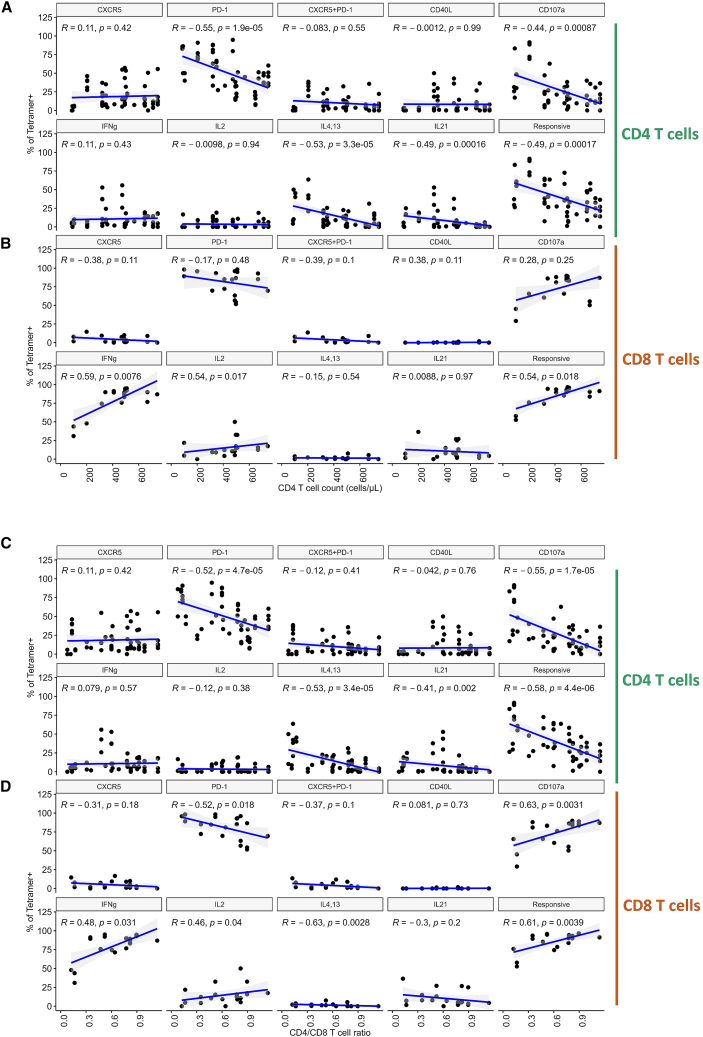
Correlations between the CD4^+^ T cell counts and the CD4^+^/CD8^+^ T cell ratio, and functional traits of CD4^+^ and CD8^+^ T cells specific for immunodominant HIV epitopes (A) Scatterplots demonstrating correlations between the CD4^+^ T cell counts and proportions of tetramer-specific CD4^+^ T cells expressing CXCR5, PD-1, CD40L, CD107a, IFN-γ, IL-2, IL-4/-13, IL-21, or any of these proteins. (B) Scatterplots demonstrating correlations between the CD4^+^ T cell counts and proportions of tetramer-specific CD8^+^ T cells expressing CXCR5, PD-1, CD40L, CD107a, IFN-γ, IL-2, IL-4/-13, IL-21, or any of these proteins. The following numbers of matched tetramer-individual combinations were included in each group: *n* = 63 for (A) and *n* = 23 for (B). (C) Scatterplots demonstrating correlations between the CD4^+^/CD8^+^ T cell ratio and proportions of tetramer-specific CD4^+^ T cells expressing CXCR5, PD-1, CD40L, CD107a, IFN-γ, IL-2, IL-4/-13, IL-21, or any of these proteins. (D) Scatterplots demonstrating correlations between the CD4^+^/CD8^+^ T cell ratio and proportions of tetramer-specific CD8^+^ T cells expressing CXCR5, PD-1, CD40L, CD107a, IFN-γ, IL-2, IL-4/-13, IL-21, or any of these proteins. The 95% confidence intervals around the line of best fit are displayed as shading. The r and *p* values are given for each line. The strength of correlations was assessed by a two–sided Spearman’s correlation test with Bonferroni’s correction for multiple testing. The following numbers of matched tetramer–individual combinations were included in each group: *n* = 63 for (A) and (C), and *n* = 23 for (B) and (D).

We observed that tetramer-specific CD4^+^ T cells expressing PD-1 (r = −0.55, *p* < 0.0001), CD107a (r = −0.44, *p* < 0.001), IL-4/IL-13 (r = −0.53, *p* < 0.0001), or IL-21 (r = −0.49, *p* < 0.001) were inversely correlated with absolute CD4^+^ T cell counts. A similar inverse correlation was also observed for the overall population of tetramer-specific CD4^+^ T cells that were responsive to stimulation with any of the analyzed markers (r = −0.49, *p* < 0.001). In contrast, for tetramer-specific CD8^+^ T cells, expression of IFN-γ (r = 0.59, *p* < 0.01), IL-2 (r = 0.54, *p* < 0.05), and overall responsiveness (r = 0.54, *p* < 0.05) showed significant positive correlations with CD4^+^ T cell counts (Figure 5A).

We furthermore analyzed relationships with the CD4/CD8 ratio. Expression of PD-1 (r = −0.52, *p* < 0.0001), CD107a (r = −0.55, *p* < 0.0001), IL-4/IL-13 (r = −0.53, *p* < 0.0001), or IL-21 (r = −0.41, *p* < 0.01) by HIV-specific CD4^+^ T cells, as well as the total population of responsive HIV-specific CD4^+^ T cells (r = −0.58, *p* < 0.0001), negatively correlated with the CD4/CD8 ratio. In contrast, tetramer-specific CD8^+^ T cells expressing CD107a (r = 0.63, *p* < 0.001), IFN-γ (r = 0.48, *p* < 0.05), IL-2 (r = 0.46, *p* < 0.05), or any activation-induced marker (r = 0.61, *p* < 0.01) showed positive correlations with the ratio. Additionally, PD-1 (r = −0.52, *p* < 0.05) and IL-4/IL-13 (r = −0.63, *p* < 0.01) expression by tetramer-specific CD8^+^ T cells was negatively correlated with the ratio of CD4^+^/CD8^+^ T cells (Figure 5B).

Taken together, these analyses reveal opposing relationships between functional HIV-specific CD4^+^ and CD8^+^ T cell subsets and clinical immunological parameters. Whereas multiple CD4^+^ T cell functional traits inversely correlated with absolute CD4^+^ T cell counts and the CD4/CD8 ratio, functional responses of HIV-specific CD8^+^ T cells generally showed positive correlations.

## Discussion

HIV infection profoundly reshapes virus-specific T cell functions through CD4^+^ T cell depletion, persistent antigenemia, and chronic activation.^1^^,^^7^ However, how the altered functional profiles of CD4^+^ and CD8^+^ subsets compare and to what extent they depend on protein specificity and the presence of other T cell functions remains unclear. Using recombinant HLA tetramers in combination with peptide stimulation, we identified T cells specific for immunodominant HIV epitopes in the peripheral blood of chronically infected individuals and characterized their frequencies, follicular access, and functional profiles. While we observed comparable frequencies of HIV-specific CD4^+^ and CD8^+^ T cells, their functional profiles diverged sharply. Protein specificity dictated CD4^+^ T cell function, and CD8^+^ T cells more frequently produced IL-2 than CD4^+^ T cells. Notably, CD8^+^ T cell functions—particularly IFN-γ and IL-2 expression—were associated with indicators of slower disease progression, whereas the effects of CD4^+^ T cells were more variable. Associations between CD4^+^ and CD8^+^ T cell functions were evident but independent of protein co-targeting.

Chronic HIV infection is characterized by progressive CD4^+^ T cell loss,^54^^,^^55^ while CD8^+^ T cells remain expanded through both antigen-driven and bystander activation.^56^^,^^57^ However, the frequencies of HIV-specific CD4^+^ and CD8^+^ T cells may equalize through concurrent antigendriven expansion of the CD4^+^ subset and activation-induced death of the CD8^+^ subset.^7^^,^^50^ Supporting this hypothesis, we observed comparable frequencies of CD4^+^ and CD8^+^ T cells specific for immunodominant HIV epitopes. Notably, p24 epitopes were highly immunogenic for both CD4^+^ and CD8^+^ T cells, consistent with the previously documented immunodominance of this relatively conserved, stable, and abundant viral protein.^30^^,^^31^^,^^32^^,^^33^^,^^34^^,^^35^^,^^36^

CXCR5 expression is critical for T cell migration into B cell follicles, which are key sites of antibody formation but also viral persistence.^58^^,^^59^ Consistent with previous reports of follicular CD8^+^ T cells during chronic viral infections,^60^^,^^61^ we detected CXCR5^+^ cells in both subsets, though expression was much more frequent among HIV-specific CD4^+^ T cells. Notably, HIV-specific CD4^+^ T cells more often co-expressed CXCR5 and PD-1, reflecting their differentiation into Tfh cells, which are essential for germinal center reactions and antibody responses^13^^,^^62^ but also implicated in maintaining HIV latency.^59^ PD-1 was expressed on a large proportion of HIV-specific T cells, particularly CD8^+^ T cells, suggesting greater activation pressure on this subset^7^ and possibly reflecting preferential depletion of activated HIV-specific CD4^+^ T cells.^63^^,^^64^ Protein specificity shaped these phenotypes only in CD4^+^ T cells. CD4^+^ T cells specific for p24 showed increased PD-1 expression in line with the high immunogenicity of the protein,^30^^,^^31^^,^^32^^,^^33^ while gp120-specific CD4^+^ T cells frequently expressed CXCR5. This might be important for the generation of neutralizing antibodies and explained by the high immunogenicity of gp120 for B cells that drive Tfh differentiation.^65^ Since all analyses were conducted on peripheral blood, circulating CXCR5^+^ T cells may only partially represent the phenotype and function of bona fide lymph node or germinal center-resident Tfh/Tfc cells, so these interpretations should be treated with caution.

Comparing the functional profiles, HIV-specific CD8^+^ T cells displayed classical cytotoxic and effector features like CD107a and IFN-γ expression, whereas HIV-specific CD4^+^ T cells more frequently expressed CD40L and IL-4/-13, molecules important for providing help to B cells. Interestingly, IL-2 production, traditionally associated with CD4^+^ T cells, was more frequently observed among HIV–specific CD8^+^ T cells. This unexpected feature is consistent with previous reports that IL-2-producing CD8^+^ T cells, although rare, are highly functional and enriched in individuals with better viral control,^66^^,^^67^ and may partly compensate for the loss of IL-2-expressing CD4^+^ T cells in HIV infection.^1^^,^^12^^,^^68^^,^^69^ Strikingly, a larger fraction of HIV-specific CD4^+^ T cells was functionally unresponsive to stimulation compared with CD8^+^ T cells, despite the more frequent PD-1 expression on CD8^+^ T cells. This supports the view that PD-1 primarily marks cell activation and does not always indicate cell exhaustion and functional senescence,^63^^,^^64^ while CD4^+^ T cell unresponsiveness may arise from preferential infection and depletion of HIV-activated CD4^+^ T cells.^55^ Protein specificity strongly shaped CD4^+^ T cell function: p24-specific CD4^+^ T cells were more cytotoxic, more responsive to stimulation, and displayed distinct polyfunctionality, consistent with the superior immunogenicity of p24.^30^^,^^31^^,^^32^^,^^33^

T cell functions are reportedly influenced by HIV viremia,^29^^,^^52^^,^^53^ while different T cell functions in turn have distinct effects on viral replication. Investigating this intricate interplay, we found that CD4^+^ T cells expressing CD107a correlated positively with viral load, which is in line with earlier studies demonstrating that viremia drives cytotoxic CD4^+^ T cell differentiation.^26^ In contrast, CD4^+^ T cells expressing IFN-γ, IL-21, or CD40L correlated inversely with viral load, consistent with their previously documented protective roles in viral control via support of CD8^+^ T cells and B cell responses.^1^^,^^11^^,^^12^^,^^13^^,^^14^^,^^15^^,^^16^^,^^17^^,^^65^^,^^70^ Among CD8^+^ T cells, not only IFN-γ and IL-2 production but also overall responsiveness correlated negatively with viral load in line with their established function in controlling HIV replication.^10^^,^^27^^,^^28^ Importantly, these interpretations rely on cross-sectional correlation analyses and prior literature and do not provide direct functional or mechanistic evidence.

We also observed multiple correlations between functional CD4^+^ and CD8^+^ T cell subsets. For instance, CD8^+^ T cells expressing CXCR5 and/or PD-1 correlated with CD4^+^ T cells producing IL-4/IL-13 as well as all stimulation-responsive CD4^+^ T cells, suggesting a role for CD4^+^ T cells in both CD8^+^ activation and follicular positioning.^7^ This is particularly relevant given that B cell follicles are a major HIV reservoir.^59^ Additionally, rare CD8^+^ T cells with helper-like features (CD40L, IL-4/IL-13) correlated with CD4^+^ T cells producing IFN-γ, CD40L, or IL-2, indicating their dependence on helper signals. Interestingly, these correlations were more frequent across responses targeting different HIV proteins than within the same protein, suggesting that CD4^+^-CD8^+^ cross-talk is largely independent of shared antigen specificity, in contrast to CD4^+^ T cell-B cell interactions.^46^^,^^47^ Overall, the correlation matrices in Figures 4C–4E are intended to illustrate the broader network of associations among functional T cell subsets and to explore potential dependence on protein co-targeting, rather than to imply causality or direct functional coupling between individual populations.

As we observed significant correlations with the viral load, we next investigated how different T cell functions correlate with further markers of disease progression. Functional traits of HIV-specific CD4^+^ T cells largely showed inverse associations with absolute CD4^+^ T cell count and the CD4/CD8 ratio. This was particularly evident for the expression of PD-1, CD107a, IL-4/13, and overall responsiveness, likely reflecting increasing virus-driven activation with disease progression, as reported previously.^8^^,^^20^^,^^26^ Surprisingly, IL-21 expression by HIV-specific CD4^+^ T cells, which correlated inversely with viral load, showed an inverse association with CD4^+^ T cell count and CD4/CD8 ratio. One possible interpretation is that IL-21-producing CD4^+^ T cells contribute to antiviral control but only expand in more advanced disease stages due to prolonged immune activation, consistent with previous findings.^71^ In contrast, HIV-specific CD8^+^ T cell functions were generally associated with slower disease progression. Expression of IFN-γ and IL-2, as well as overall responsiveness, correlated positively with CD4^+^ T cell counts and the CD4/CD8 ratio. This is in line with their inverse associations with viral load and with prior studies demonstrating the protective role of these CD8^+^ T cell functions.^67^ IL-2 is of particular interest, as our data identify HIV-specific CD8^+^ T cells as a major source of this cytokine in chronic HIV infection, potentially compensating for the loss of IL-2-producing CD4^+^ T cells.^66^ Consistent with previous reports of increased activation and exhaustion of CD8^+^ T cells during disease progression,^7^^,^^8^ PD-1 expression and expression of IL-4/-13^72^ in HIV-specific CD8^+^ T cells inversely correlated with the CD4/CD8 ratio. Taken together, these findings suggest that although HIV-specific CD4^+^ T cell functions predominantly reflect ongoing disease progression, functional CD8^+^ T cell responses are more closely associated with preserved immune status and slower progression. As noted previously, these interpretations are based on cross-sectional correlation analyses and supported by prior literature; they do not establish causality or provide direct mechanistic insight.

In conclusion, we show that during chronic HIV infection, CD4^+^ and CD8^+^ T cells recognizing immunodominant epitopes are present at comparable frequencies but differ markedly in function. CD4^+^ T cells exhibit helper-associated and protein-specific features, with p24-specificity linked to enhanced responsiveness and cytotoxicity and gp120-specificity linked to follicular homing. This highlights that the HIV proteome itself imprints distinct functions within the CD4^+^ compartment, a phenomenon not previously demonstrated. Although some CD4^+^ T cell functions correlated with lower viral load, they showed no association with slower disease progression and, in some cases, trended toward faster decline, questioning their protective role in chronic HIV infection. In contrast, CD8^+^ T cells exhibited largely protein-independent cytotoxicity and were a major source of IL-2; together with IFN-γ, these functions inversely correlated with viral load and markers of disease progression, underscoring their central role in controlling HIV. From a translational perspective, these findings indicate that future HIV vaccines and cure strategies could benefit from selectively priming CD4^+^ T cells against antigens such as p24, to enhance cytotoxic potential or gp120 to optimize antibody responses. Meanwhile, HIV-specific CD8^+^ T cells, rather than CD4^+^ T cells, may represent the primary target to boost IL-2 production and rejuvenate overall T cell immunity in HIV infection.

### Limitations of the study

Limitations of this study include the relatively small sample size for p17-specific CD4^+^ T cell responses and Nef-specific CD8^+^ T cell responses, interpretations concerning circulating CXCR5^+^ T cells that might not fully represent lymph node or germinal center Tfh/Tfc cells, and predominance of European-origin participants, which may restrict the generalizability of our findings to populations with different HLA distributions. Another limitation of the study is that some analyses are based on cross-sectional correlations, which cannot establish causality.

## Resource availability

### Lead contact

Requests for further information and resources should be directed to and will be fulfilled by the lead contact, Jernej Pušnik (jernej.pusnik@ukbonn.de).

### Materials availability

This study did not generate new unique reagents.

### Data and code availability

• The data contain information that could compromise the privacy of research participants. Data sharing restrictions imposed by national and transnational data protection laws prohibit the general sharing of data. However, upon submission of a proposal to the lead contact and approval of this proposal by (1) the principal investigator, (2) the Ethics Committee of the University of Bonn, and (3) the data protection officer of the University Hospital Bonn, data collected for the study can be made available to other researchers.
• This paper does not report original code.
• Any additional information required to reanalyze the data reported in this paper is available from the lead contact upon request.

## Acknowledgments

We thank the participants of this study who generously provided their samples. The study was supported financially by 10.13039/501100001659Deutsche Forschungsgemeinschaft (DFG) – Project number 231604701.

## Author contributions

Conceptualization, J.P. and H.S; methodology, J.P., F.M.H., and H.S.; investigation, J.P., F.M.H., A.H., E.R., and H.S; resources, J.P., S.E., and H.S.; writing – original draft, J.P.; writing – review and editing, J.P. and H.S.; funding acquisition, H.S.; supervision, H.S.

## Declaration of interests

The authors declare no competing interests.

## STAR★Methods

### Key resources table

**Table:** 

REAGENT or RESOURCE	SOURCE	IDENTIFIER
**Antibodies**		

BD FastImmune™ CD28/CD49d	BD Biosciences	Cat# 347690; RRID:AB_647457
anti–CD107a–BV711	BioLegend	Cat# 328640; RRID:AB_2565840
anti–PD–1–BUV737	BD Biosciences	Cat# 612791; RRID:AB_2870118
anti–CD8–APC–Cy7	BioLegend	Cat# 301016; RRID:AB_314134
anti–CD4–BUV805	BD Biosciences	Cat# 612912; RRID:AB_2870197
anti–IL–4–BV421	BioLegend	Cat# 500826; RRID:AB_2561679
anti–IL–13–BV421	BioLegend	Cat# 501916; RRID:AB_2616748
anti–IFNγ–BB700	BD Biosciences	Cat# 566394; RRID:AB_2744484
anti–CD40L– PE–CF594	BD Biosciences	Cat# 563589; RRID:AB_2738297
anti–IL–2–PE–Cy7	BioLegend	Cat# 500326; RRID:AB_2125593
anti–IL–21–AF647	BioLegend	Cat# 513006; RRID:AB_1227661
anti–CD3–AF700	BioLegend	Cat# 317340; RRID:AB_2563408

**Biological samples**		

27 Peripheral blood samples from HIV-infected individuals	This study	“N/A”

**Chemicals, peptides, and recombinant proteins**		

Pancoll	PAN–Biotech	Cat# P04–60500
Biotinylated recombinant HLA–peptide complexes, peptides	ImmunAware	Custom order
Streptavidin–BUV496	BD Biosciences	Cat# 564666
Streptavidin–BUV563	BD Biosciences	Cat# 565765
Streptavidin–PE	Biolegend	Cat# 405204
Streptavidin–BB515	BD Biosciences	Cat# 564453
Golgi Stop	BD Biosciences	Cat# 554724
Golgi Plug	BD Biosciences	Cat# 555029
ZombieAqua	Biolegend	Cat# 423102
CytoFix/CytoPerm Solution	BD Biosciences	Cat# 554714
Perm/Wash Buffer	BD Biosciences	Cat# 554723

**Critical commercial assays**		

QIAamp DNA Micro kit;	Qiagen	Cat# 56304

**Software and algorithms**		

BD FACSDiva™ Software Version 8.0	BD Biosciences	Cat# 659528
FlowJo 10.0.7	TreeStar	https://flowjo.com/
Sequence Pilot Software	JSI Medical Systems GmbH	https://www.jsi-medisys.de/
SPICE software, version 6.x	National Institutes of Health	https://niaid.github.io/spice/
RStudio 2021.09.0 Build 351 software	RStudio PBC	https://www.rstudio.com/

**Other**		

CompBeads	BD Biosciences	Cat# 552843
Rainbow calibration particles	Biolegend	Cat# 422905

### Experimental model and study participant details

Peripheral blood samples from 27 chronically HIV–1–infected individuals were used for this study. All participants were treatment–naïve and had a viral load ranging from 1,053,000 to 1345 copies per milliliter of blood. Age or sex was not among the selection criteria. The average age in the cohort was 46± 11 (mean ± standard deviation [SD]) years. Sex was recorded for all participants; however, the cohort was predominantly male (93%), precluding meaningful sex-stratified analyses. Further details regarding demographic and clinical characteristics of the study participants are in Table S2. Samples were collected at the Center for HIV, AIDS, and Venereal Diseases, Clinic for Dermatology, University Hospital, University Duisburg–Essen, Essen, Germany, as part of the SCABIO study investigating immunological changes in HIV–1 infection to define strategies for HIV–1 treatment, cure, and vaccination. Measurements of HIV–1 viral load were performed by the central diagnostics laboratory of University Hospital Essen using the standard clinical reverse transcription polymerase chain reaction procedure. All study participants provided written informed consent. The SCABIO study protocol was approved by the Ethics Committee of the Medical Faculty of the University of Duisburg–Essen, Germany (approval number 17–7846–BO). The institutional review board (IRB) of the University of Duisburg–Essen approved the laboratory testing and *in vitro* studies.

### Method details

#### Sample collection and storage

Study participants provided peripheral blood samples that were centrifuged for 10 min at 600 g to collect plasma. EDTA plasma was stored for further analysis at −80°C. PBMC were isolated by density gradient centrifugation using Pancoll, PAN–Biotech, P04–60500. In brief, the blood was diluted with R^+^ media (RPMI 1640 supplemented with 2 mM l–glutamine, penicillin [100 U/ml], and streptomycin [100 μg/ml]), carefully layered on top of the density gradient medium, and centrifuged at 1200 g for 10 min. The middle layer containing the PBMCs was carefully aspirated, transferred to a new tube, and washed twice with R^+^ media. Washed PBMCs were resuspended in FCS containing 10% DMSO and frozen at −80°C overnight. For long–term storage, PBMC samples were transferred to liquid nitrogen.

#### DNA isolation

The frozen content of cryopreserved PBMC samples was scraped with a lab spatula until approximately 300mg of frozen PBMC suspension was collected. From the collected material, genomic DNA was isolated using a commercial DNA purification kit (QIAamp DNA Micro kit; Qiagen) according to the manufacturer’s protocol. A concentration of eluted DNA was determined spectrophotometrically.

#### HLA genotyping

The isolated DNA samples were collected in 96–well 2D barcode–labeled tubes (Matrix™, Thermo Fisher Scientific). The study samples were combined with other samples in 96–well trays and were subsequently subjected to Next Generation Sequencing (NGS) with Illumina™ MiSeq and HiSeq platforms. For PCR amplification in–house primers were used, aiming at exons 2 and 3 for HLA class I and II alleles. Raw sequence data were analyzed by Sequence Pilot Software (JSI Medical Systems GmbH, Ettenheim, Germany), and the HLA alleles were reported using G–codes for HLA alleles that have identical nucleotide sequences across the exons encoding the peptide–binding domains.

#### Tetramerization of recombinant HLA–peptide complexes

The biotinylated recombinant HLA–peptide complexes (synthesized commercially by ImmunAware) were coupled with streptavidin–fluorophore conjugates in the following manner. First, the amount of biotinylated recombinant HLA–peptide complex needed for staining was determined and combined with the chosen streptavidin–fluorophore conjugate in a molar ratio of 12:1. The mixture was incubated for 15 min at 4°C. The addition of streptavidin–fluorophore conjugate was repeated three times, resulting in an end ratio of 3:1. The excess streptavidin–fluorophore conjugate was added to ensure that no free biotinylated recombinant HLA–peptide complexes were left in the mixture. This prevented recombining with other streptavidin–fluorophore conjugates in the staining mix and blocking of the T cell receptors. The following streptavidin–fluorophore conjugates were used: Streptavidin–BUV496 (BD Bioscience, 564666), Streptavidin–BUV563 (BD Bioscience, 565765), Streptavidin–BB515 (BD Bioscience, 564453), and Streptavidin–PE (Biolegend, 405204).

#### Staining of PBMC and flow cytometry

Cryopreserved PBMCs were thawed in R10 medium (RPMI 1640 supplemented with 10% heat–inactivated fetal calf serum, 2 mM l–glutamine, penicillin [100 U/ml], and streptomycin [100 g/ml]) and rested at 37°C and 5% CO_2_ overnight. The next morning, cells were counted, washed, and stained with a mixture of matched HLA–peptide tetramers, each at a final concentration of 40nM. At least 2 million cells were stained in each sample for 1 hour at 4°C. Following staining warm R10 supplemented with co–stimulatory antibodies (BD FastImmune™ CD28/CD49d, BD Bioscience, 347690, final concentration of 1 μg/ml), peptides present on the tetramers in soluble form (final concentration of 1 μg/ml) and anti–CD107a–BV711 (clone H4A3; Biolegend, 328640, diluted 1:200) were added to a final concentration of 1 million cells/ml. One hour into stimulation, Golgi Stop (BD Bioscience, 554724) and Golgi Plug (BD Bioscience, 555029) were added (final concentration 1 μg/ml) to inhibit vesicular transport and prevent the secretion of the cytokines from cells. Stimulation was continued for 4 more hours. Stimulated cells were then washed with PBS and subsequently stained for viability (ZombieAqua, Biolegend, 423102) for 15 min at 4°C. Next, cells were washed with FACS buffer (PBS supplemented with 2% FCS, 0.05% NaN3, and 2 mM EDTA), and a cocktail of antibodies against surface antigens was added: anti–PD–1–BUV737 (clone EH12.1; BD Bioscience, 612791, diluted 1:20), anti–CXCR5–BV785 (clone J252D4; Biolegend, 356936, diluted 1:20), and anti–CD8–APC–Cy7 (clone RPA–T8; Biolegend, 301016, diluted 1:160). After surface staining for 20 min at 4°C, cells were fixed and permeabilized in CytoFix/CytoPerm Solution (BD Bioscience, 554714) for 15 min at 4°C. Fixed cells were washed with 1x Perm/Wash Buffer (BD Bioscience, 554723), and stained for the following intracellular markers; anti–CD4–BUV805 (clone SK3; BD Bioscience, 612912, diluted 1:80), anti–IL–4–BV421 (clone MP4–25D2; Biolegend, 500826, diluted 1:20), anti–IL–13–BV421 (clone JES10–5A2; Biolegend, 501916, diluted 1:20), anti–IFNγ–BB700 (clone B27; BD Bioscience, 566394, diluted 1:10), anti–CD40L– PE–CF594 (clone TRAP–1; BD Bioscience, 563589, diluted 1:20), anti–IL–2–PE–Cy7 (clone MQ1–17H12; Biolegend, 500326, diluted 1:40), anti–IL–21–AF647 (clone 3A3–N2; Biolegend, 513006, diluted 1:80) and anti–CD3–AF700 (clone OKT3; Biolegend, 317340, diluted 1:40). Intracellular staining was performed for 20 min at 4°C. Finally, cells were washed once with Perm/Wash Buffer, twice with PBS, and acquired on a BD FACS Symphony with BD FACSDiva™ Software Version 8.0 (BD Bioscience, 659528). All antibodies and staining reagents were used in pre–titrated amounts. Fluorescence minus one (FMO) controls were included for each marker to guide gating and ensure accurate discrimination of positive from negative populations. Compensation was performed with single–stained capture beads (CompBeads, BD Bioscience, 552843), and fluctuations in laser intensity were measured every time before the experiment, using multifluorescence calibration beads (Rainbow calibration particles, Biolegend, 422905). Detection voltages were adjusted accordingly to maintain equivalent fluorescence readings throughout the experiment. Data were analyzed with FlowJo 10.0.7 (TreeStar). No technical replicates were performed due to the scarcity of the samples. The positivity cutoff for response against the tetramers was determined based on the measurements of samples stained with streptavidin–fluorochrome without the conjugated HLA–peptide complex.

### Quantification and statistical analysis

Statistical analysis was performed using RStudio 2021.09.0 Build 351 software.^73^ Differences between the groups were assessed using the Mann–Whitney test with Holm’s correction for multiple testing. All tests were performed two–sided. The strength of correlations was evaluated by Spearman’s test. Statistical significance is indicated by the following annotations: ∗p < 0.05, ∗∗p < 0.01, ∗∗∗p < 0.001, ∗∗∗∗p < 0.0001. Further statistical details of experiments can be found in the figure legends.
