# Supplementary material for: Protein-dependent CD4+ T cell specialization and CD8+ T cell-driven IL-2 production in HIV
Source: iScience. 2026 Apr 27;29(6):115895. doi: 10.1016/j.isci.2026.115895 (PMC13194185; doi:10.1016/j.isci.2026.115895)
Supplement: Document S1. Figure S1 and Tables S1 and S2 [file mmc1.pdf]

iScience, Volume *29*

Supplemental information

Protein-dependent CD4$^+$ T cell specialization and CD8$^+$ T cell-driven IL-2 production in HIV

Jernej Pušnik, Falko M. Heinemann, Andreas Heinold, Enrico Richter, Stefan Esser, and Hendrik Streeck

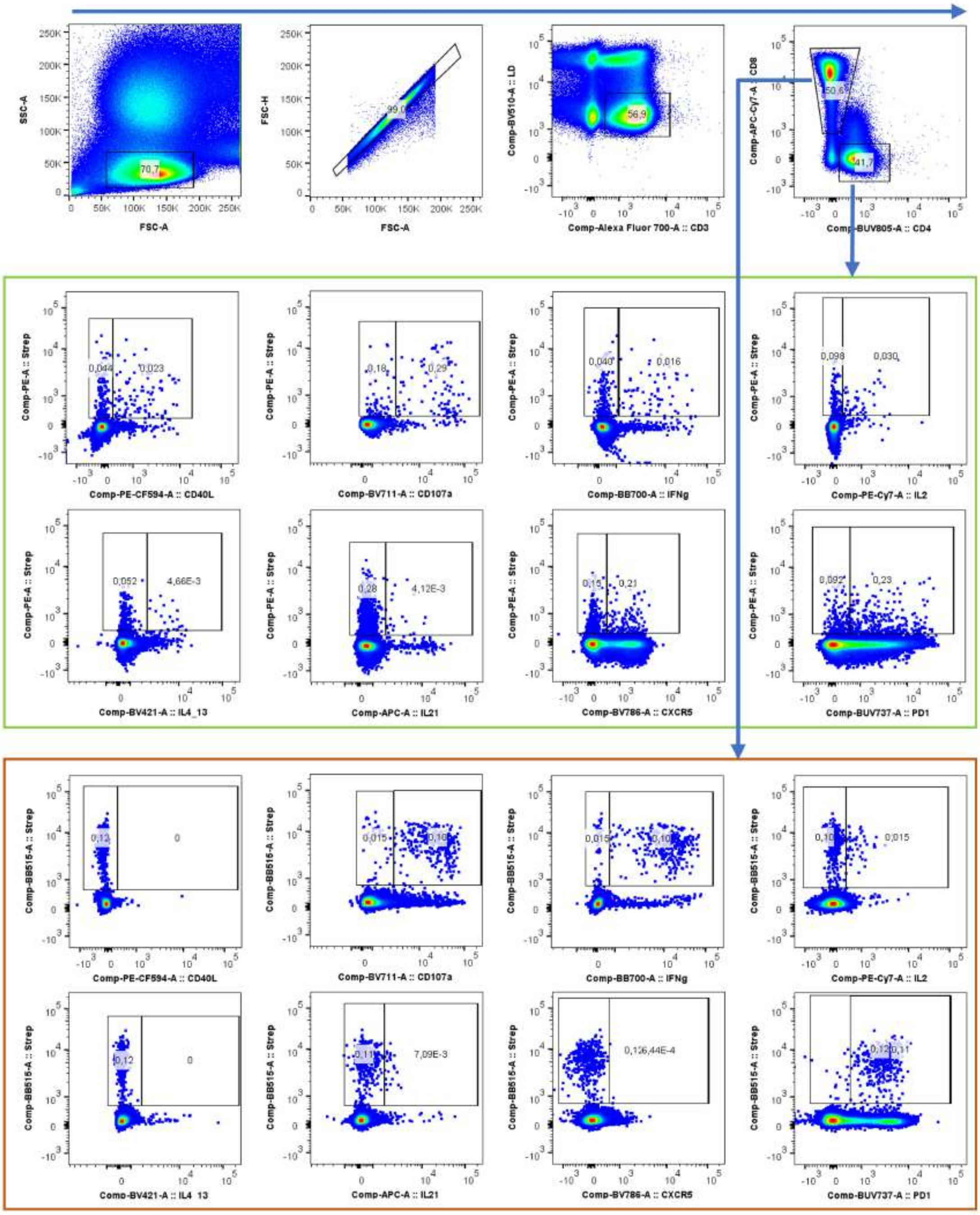

Supplemental Figure 1) Shown is the gating strategy applied for the identification of CD4 and CD8 T cells specific for immunodominant HIV epitopes in peripheral blood of treatment-naive individuals in the chronic phase of HIV infection. Peripheral blood mononuclear cells (PBMCs) were stained with fluorescent HLA–peptide tetramers, followed by stimulation with the corresponding peptides and subsequent flow cytometric staining. Plots are shown as pseudocolors. Arrows indicate the sequential order in which gates were applied. Numbers within the gates are percentages of the parent population. Axis labels indicate forward scatter (FSC), side scatted (SSC) or specificity of antibody used for staining together with a cunjugated fluorophore. „Strep“ stands for the streptavidin component of fluorescent HLA-peptide tetramers.

Sample	HLA-II-peptide-tetramer	Protein	Individual
1	DRB1*01:01_p24-1	p24	p100917
2	DRB1*01:01_p24-1	p24	p100922
3	DRB1*01:01_p24-1	p24	p830060
4	DRB1*01:01_p24-1	p24	p999653
5	DRB1*01:01_p24-4	p24	p100917
6	DRB1*01:01_p24-4	p24	p100922
7	DRB1*01:01_p24-4	p24	p830060
8	DRB1*01:01_p24-4	p24	p999653
9	DRB1*01:01_gp120-1	gp120	p100917
10	DRB1*01:01_gp120-1	gp120	p100922
11	DRB1*01:01_gp120-1	gp120	p830060
12	DRB1*01:01_gp120-1	gp120	p999653
13	DRB1*03:01_p24-2	p24	p128279
14	DRB1*03:01_p24-2	p24	p145090
15	DRB1*03:01_p24-2	p24	p174987
16	DRB1*03:01_p24-2	p24	p530089
17	DRB1*03:01_p24-2	p24	p963343
18	DRB1*07:01_p24-1	p24	p100826
19	DRB1*07:01_p24-1	p24	p100845
20	DRB1*07:01_p24-1	p24	p100863
21	DRB1*07:01_p24-1	p24	p100890
22	DRB1*07:01_p24-1	p24	p182804
23	DRB1*07:01_p24-1	p24	p530089
24	DRB1*07:01_p24-4	p24	p100826
25	DRB1*07:01_p24-4	p24	p100845
26	DRB1*07:01_p24-4	p24	p100863
27	DRB1*07:01_p24-4	p24	p100890
28	DRB1*07:01_p24-4	p24	p182804
29	DRB1*07:01_p24-4	p24	p530089
30	DRB1*11:01_p24-1	p24	p100917
31	DRB1*11:01_p24-1	p24	p182804
32	DRB1*11:01_p24-1	p24	p490271
33	DRB1*11:01_p24-1	p24	p592736
34	DRB1*11:01_p24-4	p24	p100917
35	DRB1*11:01_p24-4	p24	p182804
36	DRB1*11:01_p24-4	p24	p490271
37	DRB1*11:01_p24-4	p24	p592736
38	DRB1*13:01_p17-2	p17	p592736
39	DRB1*13:01_p17-2	p17	p903467
40	DRB1*13:01_p24-1	p24	p592736
41	DRB1*13:01_p24-1	p24	p903467
42	DRB1*13:01_p24-2	p24	p592736

43	DRB1*13:01_p24-2	p24	p903467
44	DRB1*15:01_p24-2	p24	p100826
45	DRB1*15:01_p24-2	p24	p100862
46	DRB1*15:01_p24-2	p24	p100922
47	DRB1*15:01_p24-2	p24	p145090
48	DRB1*15:01_p24-2	p24	p661372
49	DRB1*15:01_p24-2	p24	p697793
50	DRB1*15:01_p24-2	p24	p797276
51	DRB1*15:01_p24-2	p24	p866119
52	DRB1*15:01_p24-2	p24	p999653
53	DRB1*15:01_p24-3	p24	p100826
54	DRB1*15:01_p24-3	p24	p100862
55	DRB1*15:01_p24-3	p24	p100922
56	DRB1*15:01_p24-3	p24	p145090
57	DRB1*15:01_p24-3	p24	p661372
58	DRB1*15:01_p24-3	p24	p697793
59	DRB1*15:01_p24-3	p24	p797276
60	DRB1*15:01_p24-3	p24	p866119
61	DRB1*15:01_p24-3	p24	p999653
62	DRB1*15:01_gp120-2	gp120	p100826
63	DRB1*15:01_gp120-2	gp120	p100862
64	DRB1*15:01_gp120-2	gp120	p100922
65	DRB1*15:01_gp120-2	gp120	p145090
66	DRB1*15:01_gp120-2	gp120	p661372
67	DRB1*15:01_gp120-2	gp120	p697793
68	DRB1*15:01_gp120-2	gp120	p797276
69	DRB1*15:01_gp120-2	gp120	p866119
70	DRB1*15:01_gp120-2	gp120	p999653
71	DRB1*15:01_gp120-3	gp120	p100826
72	DRB1*15:01_gp120-3	gp120	p100862
73	DRB1*15:01_gp120-3	gp120	p100922
74	DRB1*15:01_gp120-3	gp120	p145090
75	DRB1*15:01_gp120-3	gp120	p661372
76	DRB1*15:01_gp120-3	gp120	p697793
77	DRB1*15:01_gp120-3	gp120	p797276
78	DRB1*15:01_gp120-3	gp120	p866119
79	DRB1*15:01_gp120-3	gp120	p999653

Sample	HLA-I-peptide-tetramer	Protein	Individual
1	A 03:01_RK9	p17	p100826
2	A 03:01_RK9	p17	p100862
3	A 03:01_RK9	p17	p100917
4	A 03:01_RK9	p17	p292139
5	A 03:01_RK9	p17	p830060
6	A 03:01_RK9	p17	p903467
7	A 03:01_RK9	p17	p963343
8	A 03:01_RK9	p17	p999653
9	A 02:01_SL9	p17	p100845
10	A 02:01_SL9	p17	p100862
11	A 02:01_SL9	p17	p100863
12	A 02:01_SL9	p17	p100890
13	A 02:01_SL9	p17	p100922
14	A 02:01_SL9	p17	p174987
15	A 02:01_SL9	p17	p243397
16	A 02:01_SL9	p17	p530089
17	A 02:01_SL9	p17	p592736
18	A 02:01_SL9	p17	p757608
19	A 02:01_SL9	p17	p277238
20	A 02:01_SL9	p17	p310166
21	B 07:02_GL9	p24	p100862
22	B 07:02_GL9	p24	p100922
23	B 07:02_GL9	p24	p100826
24	B 07:02_GL9	p24	p145090
25	B 07:02_GL9	p24	p963343
26	B 07:02_GL9	p24	p999653
27	B 08:01_FL8	Nef	p128279
28	B 08:01_FL8	Nef	p145090
29	B 08:01_FL8	Nef	p797276
30	B 08:01_EI8	p24	p128279
31	B 08:01_EI8	p24	p145090
32	B 08:01_EI8	p24	p797276
33	B 27:05_KK10	p24	p182804
34	B 57:01_TW10	p24	p100863

Supplemental Table 1: Shown are the HLA-matched tetramer-individual combinations used for identification of HIV-specific CD4+ and CD8+ T cell responses. Each combination represents one data point in the figures. The table also indicates the name of the protein where the epitope is located.

Patient	Age	Sex	Viral load (copies/mL)	CD4$^+$ T cell count (cells/ul)	CD8$^+$ T cell count (cells/ul)	CD4/CD8 ratio
1	51-60	female	197544	369	477	0,77
2	31-40	male	224700	375	512	0,73
3	31-40	male	165900	316	523	0,60
4	61-70	male	12220	497	555	0,90
5	31-40	male	11500	667	862	0,77
6	51-60	male	900300	640	2140	0,30
7	61-70	male	35571	805	1580	0,51
8	41-50	male	24628	706	719	0,98
9	21-30	female	19053	742	651	1,14
10	21-30	male	34090	409	1170	0,35
11	31-40	male	63740	489	597	0,82
12	21-30	male	21590	355	524	0,68
13	71-80	male	1053000	344	441	0,78
14	41-50	male	679700	494	704	0,70
15	41-50	male	25410	381	917	0,42
16	18-20	male	139700	562	654	0,86
17	31-40	male	18170	465	631	0,74
18	31-40	male	2248	316	535	0,59
19	41-50	male	69792	690	856	0,81
20	21-30	male	45960	643	701	0,92
21	21-30	male	1345	807	1260	0,64
22	31-40	male	78140	100	609	0,16
23	31-40	male	14050	469	996	0,47
24	31-40	male	247600	82	755	0,11
25	41-50	male	285600	87	1250	0,07
26	31-40	male	4127	508	976	0,52
27	41-50	male	636500	200	1610	0,12

Supplemental Table 2) Demographic and clinical characteristics of the study participants. The table includes the following information for each individual (columns listed from left to right): participant pseudonym, age (reported in 10-year ranges), sex, plasma HIV viral load, absolute CD4$^+$ T cell count, absolute CD8$^+$ T cell count, and the CD4/CD8 T cell ratio.
